# Polymorphism and multiple correlated characters: Do flatfish asymmetry morphs also differ in swimming performance and metabolic rate?

**DOI:** 10.1002/ece3.5080

**Published:** 2019-03-26

**Authors:** Carolyn A. Bergstrom, JoMarie Alba, Julienne Pacheco, Trevor Fritz, Sherry L. Tamone

**Affiliations:** ^1^ Biology Program, Department of Natural Sciences University of Alaska Southeast Juneau Alaska; ^2^ Department of Biological Sciences Walla Walla University College Place, Walla Walla Washington

**Keywords:** ecological selection, fast‐starts, geographical cline, Pleuronectiformes, respirometry, swimming endurance

## Abstract

1. Phenotypic polymorphisms often differ in multiple correlated traits including morphology, behavior, and physiology, all of which can affect performance. How selection acts on these suites of traits can be complex and difficult to discern. Starry flounder (*Platichthys stellatus*) is a pleuronectid flatfish that exhibits rare polymorphism for the direction of eye migration and resulting whole‐body asymmetry. *P. stellatus* asymmetry morphs differ subtly in several anatomical traits, foraging behavior, and stable isotope signatures, suggesting they may be ecologically segregated, yet performance and metabolic differences are unknown.
2. Here we tested the hypothesis that sinistral and dextral *P. stellatus* asymmetry morphs diverge in performance and routine metabolic rate (RMR) by comparing prolonged swimming endurance (time to exhaustion at a constant swimming speed), fast‐start swimming velocity and acceleration, and rate of oxygen consumption. Based on subtle morphological differences in caudal tail size, we expected sinistral *P. stellatus* to have superior prolonged swimming endurance relative to dextral fish, but inferior fast‐start performance.
3. Sinistral *P. stellatus* exhibited both significantly greater prolonged swimming performance and fast‐start swimming performance. However, sinistral *P. stellatus* also exhibited greater RMR, suggesting that their general swimming performance could be enhanced by an elevated metabolic rate.
4. Divergence between *P. stellatus* asymmetry morphs in swimming performance and metabolic rates contributes to growing evidence of ecological segregation between them, as well as our understanding of possible ecological consequences of asymmetry direction in flatfishes. These data provide an example of the complexity of polymorphisms associated with multiple correlated traits in a rare case of asymmetry polymorphism in a marine flatfish species.

## 1 INTRODUCTION

The adaptive significance of discrete morphs in polymorphic species has long been of interest in evolutionary ecology (Fisher, 1930; Maynard Smith, 1989). An emerging trend is that polymorphic species often involve divergence in multiple correlated characters (Sinervo & Svensson, 2002) and that the maintenance of morphs is due to selection on not just the primary trait(s) defining the polymorphism, but also on other correlated traits (Brodie III, 1992; Shine, Ambariyanto, Harlow, & Munpuni, 1998; Svensson, Sinervo, & Comendant, 2001). Biomechanical links between trait form and function can elucidate potential sources of selection (Wainwright, 1991). However, how selection acts on morph expression is ultimately measured by how it affects performance in a particular ecological context (Arnold, 1983; Grant, 1986; Reimchen, 1994), and performance can be mitigated by not just morphology, but behavior and physiology as well (Langerhans & Reznick, 2010). Therefore, how selection acts on an individual in a polymorphic species, a singular expression of multiple traits, can be complex and difficult to discern.

**Figure 1:**
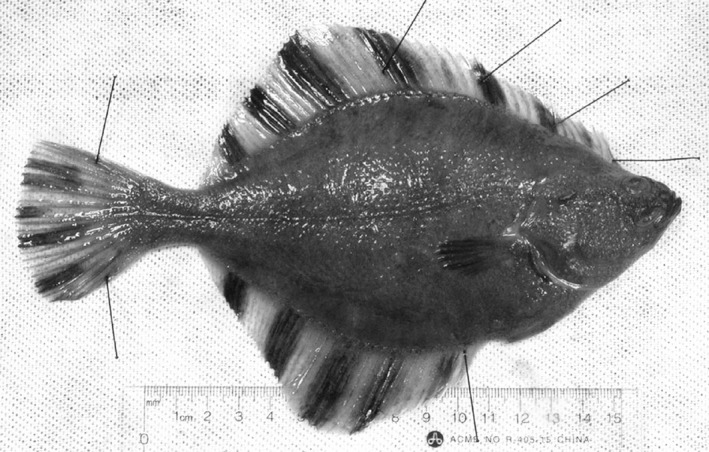
Eyed‐side view of a dextral *Platichthys stellatus*. Photograph credit: Carolyn Bergstrom

An intriguing polymorphism is seen in some flatfish species (Order Pleuronectiformes) that contain both left‐eyed (sinistral) and right‐eyed (dextral) individuals in sympatry; a rare phenomenon found in <1% of the ~800 flatfish species (Munroe, 2015). To date, it is unclear what specific intrinsic and/or extrinsic conditions lead to this polymorphism in a small number of flatfish species, while the overwhelming majority are monomorphic for eye‐sidedness. In the two species investigated in this context so far, *Platichthys flesus* and *Platichthys stellatus* (Figure 1), multiple other characters differ between sinistral and dextral individuals that suggest ecological segregation, at least trophically. Sinistral and dextral morphs in both species differ in subtle anatomical traits related to head and caudal tail shape (Bergstrom, 2007; Russo et al., 2012), and show slight shifts in diet (Bergstrom & Reimchen, 2018; Russo et al., 2012). In *P. stellatus*, there are behavioral differences during prey strikes between morphs in captive‐reared juveniles (Bergstrom & Palmer, 2007) and sinistral *P. stellatus* are slightly but significantly enriched in muscle ^15^N:^14^N ratios (an indicator of the average trophic level of their prey) among multiple northeastern Pacific samples (Bergstrom & Reimchen, 2018). Clearly, polymorphism in these flatfish species is associated with divergence of multiple characters in addition to asymmetry direction.

If and how selection acts on the suite of characters associated with polymorphism in these species is still unclear, yet at least in *P. stellatus* evidence supports the hypothesis that selection plays a role in its maintenance. First, there is moderate heritability for direction of asymmetry, where the majority of the variation in asymmetry direction in offspring is explained by parental direction phenotype (Policansky, 1982, Boklage, 1984, Bergstrom unpublished data). Some candidate genes for asymmetry direction have been identified (Hashimoto et al., 2007; Wei, Chen, Chen, & Bao, 2017) although there are likely multiple loci and environmental influences involved. Second, there is a geographical cline in the relative frequency of sinistral and dextral individuals across the species' distribution in the north Pacific (Hubbs & Kuronuma, 1942) that has persisted for at least decades (Bergstrom, 2007), suggestive of a selective environmental gradient (Endler, 1986). This, combined with evidence of ecological selection between *P. stellatus* morphs, begs the question of what the agents of selection are and what traits are targeted.

While most publications investigating functional variance in fishes focus on morphology (Langerhans & Reznick, 2010), it is ultimately performance that translates morphology into fitness consequences (Arnold, 1983). Morphological variance between *P. stellatus* asymmetry morphs do suggest potential consequences to swimming performance: dextral flounder have subtly but significantly longer and deeper caudal peduncles than sinistrals (Bergstrom, 2007). Greater caudal peduncle area among fishes is associated with improved fast‐start swimming performance because of increased thrust (Blake, 2004; Webb, 1984). Conversely, caudal surface area exhibits a functional trade‐off where fish with narrower caudal peduncles enjoy improved endurance during prolonged swimming because of reduced drag (Langerhans & Reznick, 2010; Webb, 1988), even within species (Langerhans, 2009). From this, the prediction is that dextral flounder would have superior fast‐start performance, but due to functional trade‐offs related to caudal peduncle size, sinistral flounder would have superior swimming endurance.

However, morphology is only one of many factors that can affect performance, and its effects are often mitigated by behavior and physiology (Morozov, Leinonen, Merilä, & McCairns, 2018). Metabolism in particular can exhibit considerable variation among individuals not always reflected by morphological differences (Lindholm, 2014), and recent genome scans indicate selection signals acting on metabolic variation (Marden, 2013). While variation among species in metabolic rates is well documented with respect to size, temperature, age, and physiology (Schmidt‐Nielsen, 1997), the mechanisms that generate that variation within species are not. Individuals can vary by threefold in their metabolic rate even after controlling for body mass, temperature, and age, among others (Burton, Killen, Armstrong, & Metcalfe, 2011).

Regardless of the causes of intraspecific variation in metabolic rate, it appears to have far‐reaching ecological consequences. Differences in metabolic rate can affect social status and life‐history traits in Atlantic salmon, *Salmo salar* (Metcalfe, Taylor, & Thorpe, 1995), and among fish species is positively correlated with dominance behaviors, including activity level, aggression, and boldness (Biro & Stamps, 2010). Impacts on fish swimming performance also exist, and although studies testing this effect among individuals within species are rare, findings suggest swimming performance (Metcalfe, Van Leeuwen, & Killen, 2016) and recovery after exercise (Killen, Mitchell et al., 2014; Marras, Claireaux, McKenzie, & Nelson, 2010) are positively associated with greater metabolic rates. Olive flounder (*Paralichthys olivaceus*) exhibit variation in personality, with bold flounder have greater metabolic rates and a more active swimming escape response to threats than shy flounder (Rupia, Binning, Roche, & Lu, 2016). However, to our knowledge the association between metabolic rate and swimming performance has not been demonstrated for *P. stellatus*, nor have metabolic differences between morphs been assessed in other polymorphic flatfishes.

In the current study, our objective was to gain a more comprehensive, multi‐trait understanding of phenotypic divergence between asymmetry morphs in *P. stellatus*. We tested the hypothesis that asymmetry morph is associated with both prolonged swimming endurance (Experiment 1) and fast‐start swimming performance (Experiment 2), based on subtle anatomical differences between them. Due to potential impacts on performance and fitness, we also quantified and compared routine metabolic rate between sinistral and dextral morphs (Experiment 3).

## 2 METHODS

### 2.1 General methods

#### 2.1.1 Animal collection and husbandry

Fish were collected during multiple beach‐seining trips from localities within a 65 km range near Juneau, Alaska as follows (see Table 1 for total sample sizes): Experiment 1 (April 2012) and Experiment 2 (May–July 2011) samples were from Gastineau Channel (58.340N, −134.517W); Experiment 3 (August–October 2016) samples were from Mendenhall River Estuary (58.333N, −134.604W), Eagle River Estuary (58.534N, −134.847W), and Cowee Creek Estuary (58.684N, −134.941W). Samples from three different localities for metabolic rate testing in Experiment 3 allowed us to test for spatial consistency of any detected trends.

**Table 1:** Summary statistics (mean ± 1 *SD*) of standard body length (SL) and caudal peduncle area (CA_res_) for sinistral (S) and dextral (D) starry flounder for each experiment

Experiment	Morph	SL (cm)	*t* (*p*)	CA_res_	*t* (*p*)
1 Swimming endurance (SwT)	S (*N* = 25)	13.44 ± 1.86	0.95 (0.347)	−0.13 ± 0.97	1.01 (0.317)
	D (*N* = 17)	13.96 ± 1.55		0.19 ± 1.01	
2 Fast‐start performance	S (*N* = 19)	11.66 ± 1.65	0.71 (0.481)	0.05 ± 1.14	0.14 (0.781)
	D (*N* = 19)	11.21 ± 2.22		−0.05 ± 0.84	
3 Routine metabolic rate^a^	S (*N* = 22)	10.92 ± 3.05	0.56 (0.576)	0.04 ± 0.94	0.29 (0.774)
	D (*N* = 19)	11.42 ± 3.13		−0.05 ± 1.06	

Notes

T tests show no significant differences between morphs for any morphometric trait. CA_res_ is size‐standardized. Statistics for Experiment 1 do not exclude outliers.

a Sample sizes pooled among three sample localities.

Fish were transported in coolers holding aerated seawater to the University of Alaska Southeast marine laboratory and maintained in Living Streams tanks (700‐L) with flow‐through ambient seawater (~8.8ºC). Fish were fasted for 24–48 hr before experimental trials began, and later fed pieces of herring ad libitum. Experiments were completed after six months of captivity for Experiment 1 and within one month for Experiments 2 and 3.

#### 2.1.2 Morphometrics

After each experimental trial, the eyed‐side of each fish was photographed. Standard length (SL), caudal peduncle length, and caudal peduncle depth were measured in cm from photographs of each fish using tpsDig (© F. James Rohlf). Caudal peduncle area (CA) was calculated per fish by multiplying caudal peduncle length and caudal peduncle depth. Separately for each experiment, CA was size‐standardized by saving standardized residuals from regressions of caudal area against SL. These saved residuals were used to represent size‐independent measures of caudal peduncle area (CAres).

Standard length and size‐corrected caudal area did not differ significantly from a normal distribution and so were not transformed (all Smirnov–Kolmogorov tests *p* > 0.200). The single exception was that SL in the metabolism experiment sample was bi‐modal. However, fish mass and volume were a primary variable of interest in this experiment and were corrected for each fish individually (see Metabolism experiment methods). Dextral and sinistral fish did not differ significantly in SL or CA_res_ in any of the three experiments (Table 1).

We did not measure repeatability of swimming performance or metabolic rate. However, high repeatability is reported among species of fishes for both metabolic rates (Burton et al., 2011; Metcalfe et al., 2016), and fast‐start and prolonged swimming performance (Bergstrom, 2002; Marras et al., 2010; Martínez, Guderley, Nelson, Webber, & Dutil, 2002; Morozov et al., 2018). In addition, intra‐individual variation would obscure differences between asymmetry morphs, so quantification of any detected differences would be conservative.

### 2.2 Experiment 1 Prolonged swimming performance

#### 2.2.1 Measuring prolonged swimming performance

Each fish was placed in an open system flow‐through seawater swimming chamber (66 cm length × 20 cm depth × 23 cm width), supplied with a constant flow of ambient temperature seawater. One cm above the bottom of the chamber, 10 rows of fine gauge fishing line were strung longitudinally approximately two cm apart to discourage flounder from settling on the bottom and to reduce Stephan adhesion that can affect flatfish swimming effort (Brainerd, Page, & Fish, 1997). Fish were added to the chamber with an initial water velocity of ~6.0 cm/s, and water velocity was immediately adjusted to necessitate swimming in order for fish to maintain constant horizontal position, rather than resting on the fishing lines. This was achieved within 60 s for all fish, after which water velocity was kept constant for each individual trial. Final constant water velocity was measured using a flowmeter (© SonTek FlowTracker) after completion of each trial by taking the mean of four velocity measurements at quartile lengths longitudinally in the swimming chamber, each at 60% depth and equidistant from the sides. The resulting mean final water velocity, and therefore swimming speed, was 7.67 cm/s ± 2.49 *SD*, or 1.91 ± 0.48 *SD* body lengths per second, and there were no significant differences between asymmetry morphs in either (water velocity: *t* = 0.75; *p* = 0.456, body lengths per second: *t* = 1.14; *p* = 0.261).

We measured prolonged swimming performance using the “fixed‐velocity method” (Brett, 1967; Taylor & McPhail, 1986), measured as the time in minutes each fish could maintain their horizontal position while swimming against a fixed water velocity before exhibiting signs of exhaustion. We named this variable “swim time to exhaustion,” or SwT. Fish were considered to be exhausted when they no longer maintained their horizontal position in the swim chamber, often drifting toward the back plate, and were unresponsive to brief dorso‐ventral manual pressure (similar to Wood, McMahon, & McDonald, 1977). Preferred in situ swimming speeds in other flatfish species (Kawabe, Nashimoto, Hiraishi, Naito, & Sato, 2003; Olla, Samet, & Studholme, 1972) are considerably slower than the average of 1.91 body lengths per second achieved by fish in the current study, suggesting that this velocity was challenging for them to maintain. SwT departed from a normal distribution due to a right skew (even after outliers were removed, see Section 3) and so was log_10_ transformed for all statistical analyses.

### 2.3 Experiment 2 Fast‐start swimming performance

#### 2.3.1 Measuring fast‐start performance

A 65 cm by 65 cm fiberglass tank was equipped with a Sony HDR‐CX130 video camera secured 1.8 m directly above the center of the tank. A black curtain was suspended around the perimeter of the tank to prevent visual distraction of fish. The tank was flushed and replaced with fresh seawater to a depth of 18 cm immediately prior to each trail, and a ruler was placed on the bottom for calibration.

Individual fish were placed into the tank and acclimated for five minutes. After this time, once they were stationary and facing away from the side of the tank with at least five body lengths of tank space in front of them, filming was initiated and fish were provoked to fast‐start by rapidly plunging a 2.5 cm diameter PVC pipe a few centimeters directly behind the fish's tail; a technique used successfully in other fast‐start analyses (Bergstrom, 2002; Brainerd & Patek, 1998; Harper & Blake, 1990). The fast‐start response in fishes is triggered by activation of one Mauthner cell and its spinal motor neuron pool, and is an all‐or‐nothing rather than a graded response (Eaton, Bombardieri, & Meyer, 1977); therefore it should not be dependent on stimulus intensity. All individuals were tested at an ambient water temperature of between 8–9°C.

#### 2.3.2 Video analysis

Fast‐start video footage was analyzed using iMovie 11(© Apple Inc.) and PixelStick (© Plum Amazing). For each fast‐start, the distance travelled between each video frame (30 frames per second) was measured from the tip of the snout. Measurements began immediately after the frame that exhibited the snout of the fish lifting from the tank bottom in preparation for a fast‐start. Two measures of fast‐start performance were recorded: maximum velocity (m/s) and initial acceleration (m/s^2^). Acceleration was measured only between the first two frames, and maximum velocities were captured within the first eight frames (0.27 s). Acceleration was log10 transformed due to a long right skew.

### 2.4 Experiment 3 Routine metabolic rate

#### 2.4.1 Measuring oxygen uptake rate

Oxygen consumption of individual fish was measured in a closed circuit respirometry chamber using a WITROX system (Loligo© Systems). Respirometry chambers were adapted with oxygen sensor spots coated with ruthenium to facilitate the fluorometric measurement of seawater oxygen concentration. A temperature probe continually monitored seawater temperature. Fish were placed individually into a cylindrical metabolic chamber (19.0 cm diameter × 5.0 cm height; 2.2 L total volume; Loligo© Systems) immersed in ambient seawater and allowed to acclimate for 10 min while flow‐through fresh seawater circulated through the chamber. After the acclimation period, the chamber was switched to closed recirculation mode, wherein seawater was continuously recirculated through fish‐containing metabolic chambers at ~5 L/min with an Eheim© 1046 water pump. Oxygen utilization was measured for ~10 min for each fish, and seawater oxygen saturation was never reduced below 80%.

Upon completion of each respirometry trial, individual fish were removed from the metabolic chamber, photographed for morphological measurements, and weighed to the nearest 0.01 g. The volume of seawater in the metabolic chamber was determined to take into account volume displacement of each fish and to determine the total oxygen available to the animal before and after the trial. Seawater was flushed and replenished between fish trials. Each trial produced a rate of oxygen utilization of the fish at rest that was converted to metabolic rate (mg O_2_ hr^−1^ kg^−1^). Due to the relatively short acclimation period of 10 min in the respirometry chamber before oxygen utilization measurements began, and small but inevitable spontaneous movements of the fish during trials, we refer to our metabolic rate as routine metabolic rate (RMR; Chabot, Steffensen, & Farrell, 2016) which falls between basal and active metabolic rates (Ikeda, 2016).

### 2.5 Statistical analysis

In order to test if swimming performance indices and metabolic rates differed between asymmetry morphs while controlling for body and caudal peduncle size, we ran separate ANCOVAs with each performance index or metabolic rate as the dependent, asymmetry morph as a fixed factor and SL and CA_res_ as covariates. There was no remaining correlation between size‐standardized CA_res_ and SL by definition since CA_res_ were saved residuals from a regression with SL, and residual plots of CA_res_ against SL indicated no change in variation with increasing SL. ANCOVAs were run initially with all main effects and higher‐level interaction terms included. In order to select the best model to explain each analysis, we only included interaction terms if they were significant; if interaction terms were nonsignificant, they were removed and the ANCOVA model re‐run with only main effects and significant interaction terms (where present) included. The ANCOVA for prolonged swimming performance also included water velocity as a covariate, and the ANCOVA for metabolic rate included sample location as a random factor. All data were analyzed using SPSS Statistics 22.0 program (© IBM Corporation).

## 3 RESULTS

### 3.1 Experiment 1 Prolonged swimming performance

Individual *P. stellatus* varied considerably in their swimming endurance. Two high‐endurance outliers, both sinistral, had SwTs of 25.3 and 31.2 min, more than 4.5 standard deviations from the mean of the remaining 40 fish (mean 7.38 min ± 3.94 *SD*) and almost 10 min longer than the next highest SwT, and therefore to be conservative we excluded them from further analysis. The range in SwT of the remaining fish was from 2.0 to 15.4 min. Swimming endurance (log10) was not significantly correlated with velocity of water in the flow channel in cm/s (*r* = 0.07; *df* = 38; *p* = 0.653) or in body lengths/s (*r* = 0.05; *df* = 38; *p* = 0.749).

**Figure 2:**
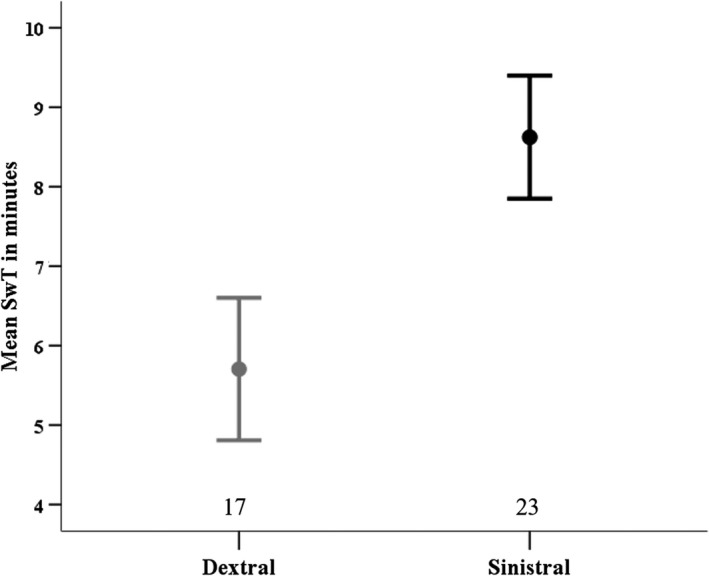
Swim time to exhaustion (SwT mean ± 1 *SE*) of sinistral (black) and dextral (gray) morphs of *P. stellatus*. Sample sizes given above x‐axis. Data graphed excludes outliers

The effect of the covariates (SL, CA_res_, water velocity) on SwT did not differ significantly between morphs (ASYMMETRY MORPH * COVARIATE interaction terms: all *F* ≤ 2.56, all *p* ≥ 0.112), nor were there significant interactions among the covariates (remaining interaction terms: all *F* ≤ 1.10; all *p* ≥ 0.305). Therefore, these interaction terms were removed from the ANCOVA to test for remaining main effects. Asymmetry morph had a significant effect on swimming endurance (Table 2) with sinistral flounder having greater mean SwT than dextrals (Figure 2). None of the other morphometric covariates (SL, CA_res_, water velocity) had a significant effect on SwT (Table 2).

**Table 2:** ANCOVAs results showing main effects of asymmetry morph, and covariates SL, CA_res_ on prolonged (Exp. 1), and fast‐start (Exp. 2) swimming performance, and (Exp. 3) routine metabolic rate

	MS	*df*	*F*	*p*
Exp. 1: SwT				
Morph	0.42	1	8.40	0.006
SL	<0.01	1	0.13	0.708
CA_res_	0.02	1	0.46	0.502
Water speed	0.01	1	0.23	0.635
Error	0.05	35		
Exp. 2: Maximum velocity				
Morph	0.36	1	9.67	0.004
SL	0.51	1	13.93	0.001
CA_res_	0.10	1	2.67	0.112
Morph*SL	0.25	1	6.70	0.014
Error	0.04	33		
Exp. 2: Initial acceleration				
Morph	0.02	1	4.69	0.038
SL	0.08	1	18.47	<0.001
CA_res_	<0.01	1	0.06	0.814
Error	<0.01	34		
Exp. 3: Routine metabolic rate				
Morph	10,366.67	1	4.56	0.040
Sample site	11,906.22	2	5.24	0.010
SL	782.61	1	0.34	0.561
CA_res_	6,977.79	1	3.07	0.089
Error	2,373.50	35		

Notes

Exp. 1 also includes water speed as a covariate, and Exp. 3 includes sample site as a random factor. All interaction terms among covariates and between covariates and asymmetry morph were nonsignificant, are not shown, and were excluded from main effects models. CA_res_ is size‐standardized. Experiment 1 excludes outliers.

### 3.2 Experiment 2 Fast‐start swimming performance

**Figure 3:**
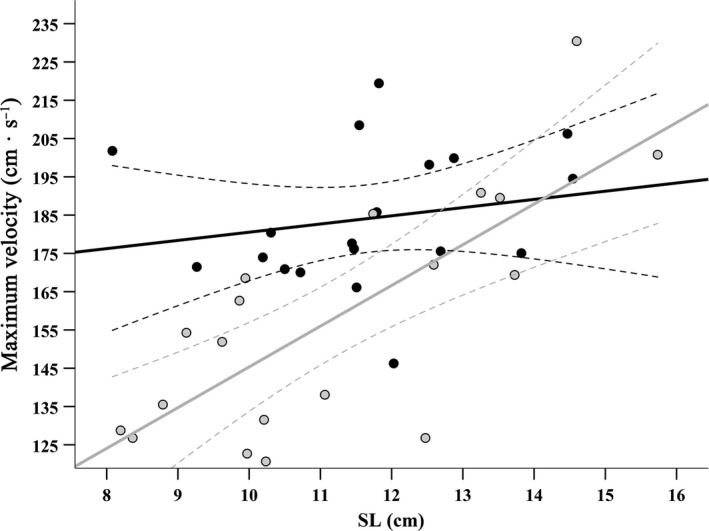
The relationship between standard length (SL) and maximum velocity for sinistral (black) and dextral (gray) morphs of *P. stellatus*. Dotted lines are 95% confidence intervals for each regression slope

Maximum velocities ranged from 1.21 to 2.30 m/s among *P. stellatus* individuals (mean = 1.71 ± 028 *SD*). Asymmetry morph and SL both had a significant effect on maximum velocity, with sinistral flounder achieving greater velocities than dextral flounder, and velocity increasing with SL (Table 2). However, maximum velocity increased with SL at a greater rate for dextral than sinistral flounder (Figure 3) as indicated by a significant SL*MORPH interaction term (Table 2), meaning that the difference in velocity between morphs decreased among the larger juveniles. Both the interaction between asymmetry morph and CA_res_ (*F* = <0.01; *p* = 0.956), and between SL and CA_res_ (*F* = 1.56; *p* = 0.221) were nonsignificant and so were removed from the final model. There was no significant main effect of CA_res_ on velocity.

**Figure 4:**
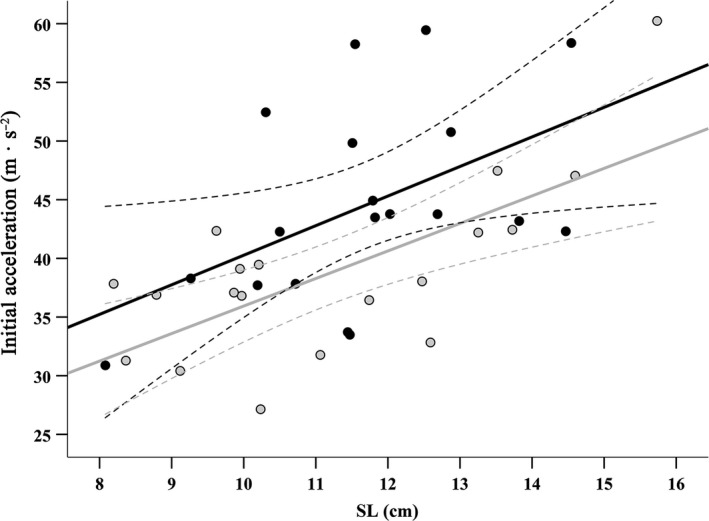
The relationship between standard length (SL) and initial acceleration for sinistral (black) and dextral (gray) morphs of *P. stellatus*. Dotted lines are 95% confidence intervals for each regression slope

Initial acceleration ranged considerable among individuals from 27.14 to 60.23 m/s^2^ (mean = 41.62 ± 8.40 *SD*). Initial acceleration was affected by SL and CA_res_ similarly in both asymmetry morphs, so these nonsignificant interaction terms between the covariates and asymmetry morph (all *F* ≤ 0.09; all *p* ≥ 0.761) were excluded from the ANCOVA, as was the nonsignificant interaction between covariates (SL*CA_res_: *F* = 0.32; *p* = 0.577). Like maximum velocity, initial acceleration was significantly greater in sinistral fish than dextral fish and increased significantly with SL (Table 2, Figure 4), but was not significantly affected by CA_res_.

### 3.3 Experiment 3 Routine metabolic rate (RMR)

**Figure 5:**
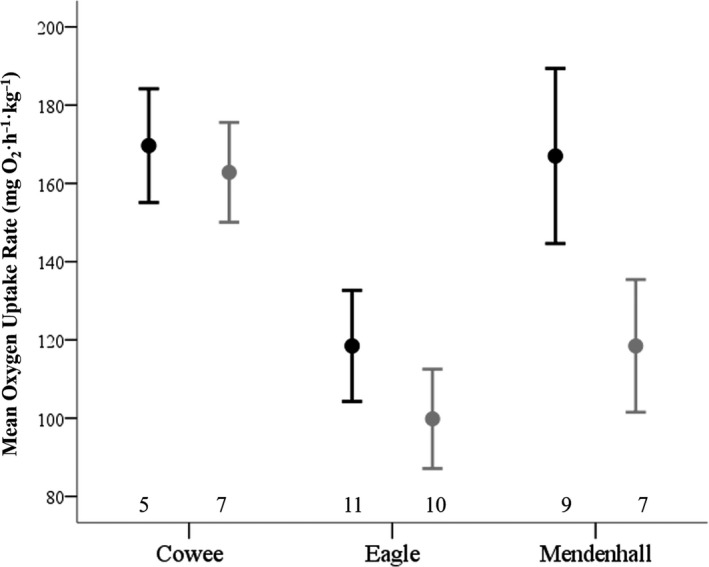
RMR as estimated by oxygen uptake rate (mean ± 1 *SE*) of sinistral (black) and dextral (gray) morphs of *P. stellatus* among three sample sites. Sample sizes given above *x*‐axis

RMR ranged from 38.57 to 292.67 mg O_2_ hr^−1^ kg^−1^ (mean = 135.14 ± 52.80 *SD*). The relationship between morphology (SL, CA_res_) and RMR did not differ significantly between morphs (interaction terms of covariates and asymmetry morph: all *F* ≤ 0.41, all *p* ≥ 0.668) nor was there a significant interaction term between morph and sample site (*F* = 0.51; *p* = 0.610) or between SL and CA_res_ (*F* = 0.03, *p* = 0.871). Therefore, the interaction terms were removed from the ANCOVA to test for main effects of asymmetry morph, sample site, and morphometric variables. Asymmetry morph had a weak but significant effect on RMR (Table 2) with sinistral flounder having slightly greater O_2_ consumption rates than dextral flounder (Figure 5). Overall, RMR differed significantly among our sample sites, but the difference in RMR between morphs did not vary significantly among sample sites as evident by the nonsignificant interaction term between morph and site. RMR was not affected significantly by SL or CA_res_ (Table 2).

## 4 DISCUSSION

Polymorphism in asymmetry direction in *Platichthys* spp. is associated with divergence in multiple anatomical (Bergstrom, 2007; Russo et al., 2012), behavioral (Bergstrom & Palmer, 2007), and trophic (Bergstrom & Reimchen, 2018; Russo et al., 2012) characters, and our current results regarding swimming performance and RMR in *P. stellatus* are consistent with this trend. Sinistral *P. stellatus* out‐performed dextrals in swimming endurance and fast‐start swimming performance, and also had slightly elevated routine metabolic rates. While we did not use the same fish in each experiment and so cannot assess direct correlations between performance and metabolism, differences in each add to the suite of divergent characters between morphs with plausible ecological and fitness consequences.

Sinistral flounder had considerably greater prolonged swimming endurance than dextrals, swimming on average 8.6 min before fatiguing, compared to 5.7 min for dextral flounder. This is roughly comparable to the only other published documentations of prolonged swimming performance in *P. stellatus* to our knowledge, which induced exhaustion in all fish after 10 min of repeatedly chasing them in a tank (Milligan & Wood, 1987; Wood et al., 1977). However, in the current study 12 of the 40 fish tested had SwTs of 10 min or greater. This greater endurance is not surprising given that the technique used in these previous studies would have induced repeated fast‐starts, likely imposing a greater metabolic load than would steady swimming at one speed.

Greater endurance in sinistral *P. stellatus* is consistent with reports of their relatively smaller caudal peduncles (Bergstrom, 2007), given the common trade‐off between steady and fast‐start swimming due to variation in caudal size (Langerhans & Reznick, 2010; Webb, 1988). However, the asymmetry morphs used in the current study did not differ significantly in their caudal peduncle size, nor was caudal peduncle size a predictor of swimming endurance. There may be other morphological attributes that contribute to steady swimming performance in this and other species of flatfish, which in addition to caudal propulsion use undulations of their median fins to propel themselves, and a “tailbeat‐and‐glide” technique over longer distances (Gibson, Stoner, & Ryer, 2015; Orcutt, 1950).

While hypotheses about the fitness consequences of swimming endurance abound, there are relatively few specific empirical examples that demonstrate it (Langerhans & Reznick, 2010; Plaut, 2001) especially within species (Marras et al., 2010). However, greater swimming endurance is associated with migratory capacity (Taylor & McPhail, 1986), maintenance of position in flowing water (Blake, 2004; Peake, McKinley, & Scruton, 2005), and greater success in prolonged cruising pursuit of prey (Rice & Hale, 2010), not to mention improved escapement from fishing trawls (Winger, He, & Walsh, 1999). While primarily demersal, some flatfishes do spend time engaged in prolonged above‐benthos swimming, particularly at night (Hunter, Metcalfe, O'Brien, Arnold, & Reynolds, 2004; Kawabe et al., 2009; Walsh & Morgan, 2004). Adult *P. stellatus* migrate from nearshore spawning grounds to deeper waters on the continental shelf annually (Orcutt, 1950), although it is unknown if longer migrations occur. While the conspecific *P. flesus* exhibits strong spawning site fidelity, tagged adults have been recaptured up to 170 km (Dando, 2011) and 360 km (Hartley, 1940) from the release site. In addition, *P. stellatus* juveniles frequently migrate up streams (Personal observation; Morrow, 1980) and have been captured 75 miles up the Columbia River (Gunter, 1942). Therefore, prolonged swimming endurance is likely to be beneficial for *P. stellatus* during even short migrations or while in stream habitats, and based on our results, sinistral individuals may enjoy a performance advantage over dextrals in these contexts. Greater prolonged swimming endurance in sinistral *P.stellatus*, if also present in the wild, may be one factor contributing to their broader geographical range across the north Pacific (Bergstrom, 2007; Hubbs & Kuronuma, 1942).

Sinistral *P. stellatus* also exhibited greater fast‐start performance than dextrals, although the difference between morphs was more subtle than that for swimming endurance (Table 2), and differences in velocity were somewhat dependent on SL (Figure 3). Nonetheless, even subtle differences in timing are important in predator–prey interactions: fishes with greater fast‐start velocities increase chances of avoiding predators during the pursuit phase (Katzir & Camhi, 1993; Walker, Ghalambor, Griset, McKenney, & Reznick, 2005), as well as increase chances of catching prey, although both interactions are also affected by factors such as water clarity (Reimchen, Bergstrom, & Nosil, 2013) and reaction distance (Domenici & Blake, 1997). Stomach contents of sinistral *P. stellatus* contain slightly greater trophic diversity than dextral fish and have elevated ^15^N:^14^N isotopic ratios in their muscle tissue (Bergstrom & Reimchen, 2018), suggesting they target prey of slightly higher trophic levels. This pattern is not exclusive to *P. stellatus*, as a sample of the congeneric European flounder (*P. flesus*) also had significant differences in stomach contents between asymmetry morphs (Russo et al., 2012). Increases in velocity and acceleration in sinistral flounder could generate trophic segregation from dextrals if prey items also differ in their own startle response performance; a likely scenario with the relatively wide taxonomic range of *P. stellatus* prey.

Given the correlation between caudal peduncle size and fast‐start performance across species (Blake, 2004; Webb, 1984), and the larger peduncles in dextral *P. stellatus* in some populations (Bergstrom, 2007), we were surprised to find faster starts in sinistral flounder. Flatfishes do use rapid propulsive thrusts of their caudal tail during fast‐starts (Orcutt, 1950), although the correlation between caudal size and fast‐start performance has not been demonstrated in any flatfish species.

Other factors besides caudal peduncle size are likely to affect fast‐start performance in flatfishes. In the current study, total body size affected both maximum velocity and initial acceleration, consistent with the widespread association between body size and swimming performance in fishes (Bainbridge, 1958; Webb, 1976). Additionally, the swimming technique of flatfishes is particularly unique due to the 90° rotation of their body and resulting orientation of other fins used for locomotion (Gibson et al., 2015). Most species of flatfishes spend considerable time resting on the benthos, and initiate fast‐starts when in contact with a physical surface of some sort (Brainerd et al., 1997). This is in marked contrast to other fishes that initiate fast‐starts with only water on both sides of their bodies, even those that rest with their ventral surface on the substrate. Lateral ground contact can affect flatfish fast‐starts because the relative hardness of the solid substrate compared to water converts more muscle energy into motion by preventing body recoil (Webb, 1981). Ultimately, the current study does show that sinistral and dextral *P. stellatus *diverge in several aspects of swimming performance due to factors independent of caudal peduncle size. While there could be other morphological attributes that vary between morphs that influence fast‐start performance against a hard surface, it is possible that the classic “morphology→performance→fitness” approach (Arnold, 1983) in *P. stellatus* also includes nonmorphological traits such as behavior and metabolism (Langerhans & Reznick, 2010).

Our results show that metabolic rates differ between asymmetry morphs. Sinistral *P. stellatus *had greater RMR than dextrals, and although the difference was subtle, it did not differ significantly among three sampled localities (Figure 5). Increased RMR is associated with improved swimming performance, faster recovery after exertion, behavioral boldness, and more active predator escape responses in other fishes (Killen, Mitchell et al., 2014; Marras et al., 2010; Metcalfe et al., 2016; Rupia et al., 2016; Yan, He, Cao, & Fu, 2013). Therefore, subtle RMR increases in sinistral *P. stellatus* could influence their swimming performance and trophic interactions and might be one factor leading to trophic segregation between morphs (Bergstrom & Reimchen, 2018).

Metabolic rate could also be associated with a temperature gradient across the range of *P. stellatus*, which occurs in the north Pacific from central California to Russia, Japan, and the Korean peninsula. The temperature gradient from California to Alaska aligns with a cline in asymmetry morph frequency, from 50% sinistral fish in California to 100% sinistral fish at the western edge of the Aleutian Islands and along the Russian and Asian coast (Hubbs & Kuronuma, 1942). *P. stellatus* from Alaska express elevated gene dosage and resulting blood circulation of anti‐freeze protein AFP‐1 compared to fish from California (Nabeta, 2009), suggesting adaptive responses to decreasing temperatures have occurred. The positive correlation between metabolic rate and temperature in ectotherms, including flatfishes (Fonds, Cronie, Vethaak, & Van der Puyl, 1992), predicts that *P. stellatus* RMR at the 8.5°C in our experiments will be greater than in fish from colder, higher latitudes, and colder temperatures reduce swimming performance in ectothermic fishes (Johnson, Cullum, & Bennett, 1993; Webb, 1978; Winger et al., 1999). Therefore, fitness advantages of slightly elevated RMR in sinistral fish might be greater in colder water, insofar as it improves swimming performance. Assuming that there is a linear relationship between RMR and latitude, and the nonsignificant interaction term between morph and RMR in the present study holds true across larger geographical ranges, this may partially explain the greater frequency of sinistral *P. stellatus* with increased latitude and decreased temperatures along the coast of North America.

The average RMR of *P. stellatus* in our study was 134.04 mg O_2_ hr^−1^ kg^−1^; comparable to those found across fish taxa (Bond, 2007; Ikeda, 2016), but relatively high compared to reports from other flatfishes (Duthie, 1982; Priede & Holliday, 1980). One explanation is that our flounder were juveniles, all 16.0 cm SL or less, while flatfish used in these other studies were adults of ~30.0 cm SL. Higher rates in our smaller fish is not surprising given the negative correlation between mass‐specific metabolic rates and body size across taxa. Another explanation is that our acclimation period was not long enough to allow for fish to completely settle after being captured and transferred between tanks (Chabot et al., 2016). However, given that all of our fish were treated in the same way, and that we are primarily concerned with differences between morphs rather than absolute rate values, slightly elevated metabolic rates across all of our fishes is not a concern.

It is not clear what could be generating metabolic differences between morphs in *P.stellatus*. A range of factors are associated with intraspecific variation in metabolic rates in fishes including genetic differences, maternal effects, parasite load, and growth rates (Burton et al., 2011), although some of these can both cause and be affected by differences in metabolic rate. It seems unlikely that simply being sinistral or dextral would directly impact metabolic rate, but there may be indirect mechanisms involved if asymmetry direction has even subtle effects on diet or locomotion.

It is possible that nitrogen isotope ratios are affected by metabolic rates, and a previous study found slightly elevated ^15^N:^14^N isotope ratios in sinistral starry flounder (Bergstrom & Reimchen, 2018). If fish with slower metabolic rates are converting relatively more of their nitrogen into growth rather than respiration, this may lead to less turnover and resynthesis of amino acids, fewer opportunities for isotopic fractionation, and thus lower ^15^N:^14^N isotope ratios (McMahon & McCarthy, 2016). This is consistent with the combination of lower ^15^N:^14^N, reduced swimming performance, and slower metabolic rate in dextral *P. stellatus*. While we have not tested in the current study for differences in growth rate, an hypothesis is that dextral individuals may be conserving the costs associated with greater speed and investing in growth rather than elevated activity, a trade‐off seen in other fishes (Killen, Marras, & McKenzie, 2014).

Flatfishes are gaining interest as a fascinating system with which to study the genetic and developmental mechanisms of whole‐body asymmetry (Hashimoto et al., 2007; Shao et al., 2017) as well as its evolutionary origin (Friedman, 2008; Harrington et al., 2016). The study of relationships between morphology, performance, physiology, and ecological selection complements these approaches and ultimately contribute to understanding the success of this order and the variation among flatfish species in their novel body asymmetry. Within some flatfish species, the emerging picture is one of a polymorphism conspicuously defined by whole‐body asymmetry direction, but which encompasses multiple and less obvious characters—morphology, behavior, oxygen consumption rate, and swimming performance—that are correlated with direction. This presents a classic challenge (Lande & Arnold, 1983) of identifying selective agents and their relative strength on a suite of correlated traits.

## CONFLICT OF INTEREST

None declared.

## AUTHORS' CONTRIBUTIONS

CAB and SLT conceived the ideas and designed the methodology; JA, TF, and JP collected the data; CAB analyzed the data; CAB and SLT led the writing of the manuscript.

## Data Availability

Dryad: https://doi.org/10.5061/dryad.cm1h0p6.

## References

[ece35080-bib-0001] Arnold, S. (1983). Morphology, performance and fitness. American Zoologist, 23, 347–361. 10.1093/icb/23.2.347

[ece35080-bib-0002] Bainbridge, R. (1958). The speed of swimming fish as related to body size and to the frequency and amplitude of the tail beat. Journal of Experimental Biology, 35, 109–133.

[ece35080-bib-0003] Bergstrom, C. A. (2002). Fast‐start swimming performance and reduction in lateral plate number in threespine stickleback. Canadian Journal of Zoology, 80, 207–213. 10.1139/z01-226

[ece35080-bib-0004] Bergstrom, C. A. (2007). Morphological evidence of correlational selection and ecological segregation between dextral and sinistral forms in a polymorphic flatfish, *Platichthys stellatus* . Journal of Evolutionary Biology, 20, 1104–1114. 10.1111/j.1420-9101.2006.01290.x 17465920

[ece35080-bib-0005] Bergstrom, C. A. , & Palmer, A. R. (2007). Which way to turn? Effect of direction of body asymmetry on turning and prey strike orientation in starry flounder *Platichthys stellatus* (Pallas) (Pleuronectidae). Journal of Fish Biology, 71, 737–748. 10.1111/j.1095-8649.2007.01531.x

[ece35080-bib-0006] Bergstrom, C. A. , & Reimchen, T. E. (2018). Isotopic trophic segregation associated with asymmetry direction in a polymorphic flatfish, *Platichthys stellatus* (Pleuronectiformes: Pleuronectidae). Biological Journal of the Linnean Society, 123, 754–766. 10.1093/biolinnean/bly004

[ece35080-bib-0007] Biro, P. A. , & Stamps, J. A. (2010). Do consistent individual differences in metabolic rate promote consistent individual differences in behavior? Trends in Ecology and Evolution, 25, 653–659. 20832898 10.1016/j.tree.2010.08.003

[ece35080-bib-0008] Blake, R. W. (2004). Fish functional design and swimming performance. Journal of Fish Biology, 65, 1193–1222. 10.1111/j.0022-1112.2004.00568.x 20738559

[ece35080-bib-0009] Boklage, C. E. (1984). On the inheritance of directional asymmetry (sidedness) in the starry flounder, *Platichthys stellatus*: Additional analyses of Policansky's data. Behavioral and Brain Sciences, 7, 725–730. 10.1017/S0140525X00028326

[ece35080-bib-0010] Bond, M. B. (2007). Bond's biology of fishes, 3rd ed Pacific Grove, CA: Thomson Brook/Cole.

[ece35080-bib-0011] Brainerd, E. L. , Page, B. N. , & Fish, F. E. (1997). Opercular jetting during fast‐starts by flatfishes. Journal of Experimental Biology, 200, 1179–1188. 9319024 10.1242/jeb.200.8.1179

[ece35080-bib-0012] Brainerd, E. L. , & Patek, S. N. (1998). Vertebral column morphology, c‐start curvature, and the evolution of mechanical defenses in tetraodontiform fishes. Copeia, 1998, 971–984. 10.2307/1447344

[ece35080-bib-0013] Brett, J. R. (1967). Swimming performance of sockeye salmon (*Oncorhynchus nerka*) in relation to fatigue time and temperature. Journal of the Fisheries Research Board of Canada, 24, 1731–1741.

[ece35080-bib-0014] Brodie III, E. D. (1992). Correlational selection for colour pattern and antipredator behavior in the garter snake *Thamnophis ordinoides* . Evolution, 46, 1284–1298. 28568995 10.1111/j.1558-5646.1992.tb01124.x

[ece35080-bib-0015] Burton, T. , Killen, S. S. , Armstrong, J. D. , & Metcalfe, N. B. (2011). What causes intraspecific variation in resting metabolic rate and what are its ecological consequences? Proceedings of the Royal Society of London B, 278, 3465–3473. 10.1098/rspb.2011.1778 PMC3189380 21957133

[ece35080-bib-0016] Chabot, D. , Steffensen, J. F. , & Farrell, A. P. (2016). The determination of standard metabolic rate in fishes. Journal of Fish Biology, 88, 81–121. 10.1111/jfb.12845 26768973

[ece35080-bib-0017] Dando, P. R. (2011). Site fidelity, homing and spawning migrations of flounder *Platichthys flesus* in the Tamar estuary, South West England. Marine Ecology Progress Series, 430, 183–196. 10.3354/meps09116

[ece35080-bib-0018] Domenici, P. D. , & Blake, R. W. (1997). The kinematics and performance of fish fast‐start swimming. Journal of Experimental Biology, 200, 1165–1178. 9319004 10.1242/jeb.200.8.1165

[ece35080-bib-0019] Duthie, G. G. (1982). The respiratory metabolism of temperature‐adapted flatfish at rest and during swimming activity and the use of anaerobic metabolism at moderate swimming speeds. Journal of Experimental Biology, 97, 359–373. 7086347 10.1242/jeb.97.1.359

[ece35080-bib-0020] Eaton, R. C. , Bombardieri, R. A. , & Meyer, D. L. (1977). The Mauthner‐initiated startle response in teleost fish. Journal of Experimental Biology, 66, 65–81. 870603 10.1242/jeb.66.1.65

[ece35080-bib-0021] Endler, J. A. (1986). Natural selection in the wild. Princeton, NJ: Princeton University Press.

[ece35080-bib-0022] Fisher, R. A. (1930). The genetical theory of natural selection. Oxford, UK: Oxford University Press.

[ece35080-bib-0023] Fonds, M. , Cronie, R. , Vethaak, A. D. , & Van der Puyl, P. (1992). Metabolism, food consumption and growth of plaice (*Pleuronectes platessa*) and flounder (*Platichthys flesus*) in relation to fish size and temperature. Netherlands Journal of Sea Research, 29, 127–143. 10.1016/0077-7579(92)90014-6

[ece35080-bib-0024] Friedman, M. (2008). The evolutionary origin of flatfish asymmetry. Nature, 454, 209–212. 10.1038/nature07108 18615083

[ece35080-bib-0025] Gibson, R. N. , Stoner, A. W. , & Ryer, C. H. (2015). The behavior of flatfishes In Gibson R. N., Nash R. D. M., Geffen A. J., & Van der Veer H. W. (Eds.), Flatfishes: Biology and exploitation (pp. 314–345). West Sussex, UK: Wiley Blackwell.

[ece35080-bib-0026] Grant, P. R. (1986). Ecology and evolution of Darwin's finches. Princeton, NJ: Princeton University Press.

[ece35080-bib-0027] Gunter, G. (1942). A list of the fishes of the mainland of North and Middle America recorded from both freshwater and sea water. The American Midland Naturalist, 28, 305–326. 10.2307/2420818

[ece35080-bib-0028] Harper, D. G. , & Blake, R. W. (1990). Fast‐start performance of rainbow trout *Salmo gairdneri* and northern pike *Esox lucius* . Journal of Experimental Biology, 150, 321–342.

[ece35080-bib-0029] Harrington, R. C. , Faircloth, B. C. , Eytan, R. I. , Smith, W. L. , Near, T. J. , Alfaro, M. E. , & Friedman, M. (2016). Phylogenomic analysis of carangimorph fishes reveals flatfish asymmetry arose in a blink of the evolutionary eye. BMC Evolutionary Biology, 16, 224 10.1186/s12862-016-0786-x 27769164 PMC5073739

[ece35080-bib-0030] Hartley, P. H. T. (1940). The Saltash tuck‐net fishery and the ecology of some estuarine fishes. Journal of the Marine Biology Association of the UK, 24, 4772–68. 10.1017/S0025315400054448

[ece35080-bib-0031] Hashimoto, H. , Aritaki, M. , Uozumi, K. , Uji, S. , Kurokawa, T. , & Suzuki, T. (2007). Embryogenesis and expression profiles of *charon* and Nodal‐pathway genes in sinistral (*Paralichthys olivaceus*) and dextral (*Verasper variegatus*) flounders. Zoological Science, 24, 137–146. 17409727 10.2108/zsj.24.137

[ece35080-bib-0032] Hubbs, C. L. , & Kuronuma, K. (1942). Hybridization in nature between two genera of flounders in Japan. Papers of the Michigan Academy of Science, 27, 267–306.

[ece35080-bib-0033] Hunter, E. , Metcalfe, J. D. , O'Brien, C. M. , Arnold, G. P. , & Reynolds, J. D. (2004). Vertical activity patterns of free‐swimming adult plaice in the southern North Sea. Marine Ecology Progress Series, 279, 261–273. 10.3354/meps279261

[ece35080-bib-0034] Ikeda, T. (2016). Routine metabolic rates of pelagic marine fishes and cephalopods as a function of body mass, habitat temperature and habitat depth. Journal of Experimental Marine Biology and Ecology, 480, 74–86. 10.1016/j.jembe.2016.03.012

[ece35080-bib-0035] Johnson, T. J. , Cullum, A. J. , & Bennett, A. F. (1993). The thermal dependence of C‐start performance in fish: Physiological versus biophysical effects. American Zoologist, 33, 65A.

[ece35080-bib-0036] Katzir, G. , & Camhi, J. M. (1993). Escape response of black mollies (*Poecilia shenops*) to predatory dives of a pied kingfisher (*Ceryl rudis*). Copeia, 1993, 549–553.

[ece35080-bib-0037] Kawabe, R. , Nashimoto, K. , Hiraishi, T. , Naito, Y. , & Sato, K. (2003). A new device for monitoring the activity of freely swimming flatfish, Japanese flounder *Paralichthys olivaceus* . Fisheries Science, 69, 3–10. 10.1046/j.1444-2906.2003.00581.x

[ece35080-bib-0038] Kawabe, R. , Yoshiura, N. , Nashimoto, K. , Tsuda, Y. , Kojima, T. , Takagi, T. , … Naito, Y. (2009). High‐frequency depth recording reveals the vertical movement of flounder in the Tsugaru Strait of northern Japan. Marine and Freshwater Behaviour and Physiology, 42, 275–295. 10.1080/10236240903169255

[ece35080-bib-0039] Killen, S. S. , Marras, S. , & McKenzie, D. J. (2014). Fast growers spring slower: Effects of food deprivation and re‐feeding on sprint swimming performance in individual juvenile European sea bass. Journal of Experimental Biology, 217, 859–865. 24265431 10.1242/jeb.097899

[ece35080-bib-0040] Killen, S. S. , Mitchell, M. D. , Rummer, J. L. , Chivers, D. P. , Ferrari, M. C. O. , Meekan, M. G. , & McCormick, M. I. (2014). Aerobic scope predicts dominance during early life in a tropical damselfish. Functional Ecology, 28, 1367–1376. 10.1111/1365-2435.12296

[ece35080-bib-0041] Lande, R. , & Arnold, S. J. (1983). The measurement of selection on correlated characters. Evolution, 37, 1210–1226. 10.1111/j.1558-5646.1983.tb00236.x 28556011

[ece35080-bib-0042] Langerhans, R. B. (2009). Trade‐off between steady and unsteady swimming underlies predator‐driven divergence in *Gambusia affinis* . Journal of Evolutionary Biology, 22, 1057–1075. 21462405 10.1111/j.1420-9101.2009.01716.x

[ece35080-bib-0043] Langerhans, R. B. , & Reznick, D. N. (2010). Ecology and evolution of swimming performance in fishes: Predicting evolution with biomechanics In Domenici P., & Kapoor B. G. (Eds.), Fish locomotion: An eco‐ethological perspective (pp. 200–248). Enfield, NH: Science Publishers.

[ece35080-bib-0044] Lindholm, M. (2014). Morphologically conservative but physiologically diverse: The mode of stasis in Anostraca (Crustacea: Branchiopoda). Evolutionary Biology, 41, 503–507. 10.1007/s11692-014-9283-6 25152547 PMC4129224

[ece35080-bib-0045] Marden, J. H. (2013). Nature's inordinate fondness for metabolic enzymes: Why metabolic enzyme loci are so frequently targets of selection. Molecular Ecology, 22, 5743–5764. 10.1111/mec.12534 24106889

[ece35080-bib-0046] Marras, S. , Claireaux, G. , McKenzie, D. J. , & Nelson, J. A. (2010). Individual variation and repeatability in aerobic and anaerobic swimming performance of European sea bass, *Dicentrarchus labrax* . Journal of Experimental Biology, 213, 26–32. 10.1242/jeb.032136 20008358

[ece35080-bib-0047] Martínez, M. , Guderley, H. , Nelson, J. A. , Webber, D. , & Dutil, J. D. (2002). Once a fast cod, always a fast cod: Maintenance of performance hierarchies despite changing food availability in cod (*Gadus morhua*). Physiological and Biochemical Zoology, 75, 90–100. 11880982 10.1086/339213

[ece35080-bib-0048] Maynard Smith, J. (1989). Evolutionary genetics. Oxford, UK: Oxford University Press.

[ece35080-bib-0049] McMahon, K. W. , & McCarthy, M. D. (2016). Embracing variability in amino acid *δ* ^15^N fractionation: Mechanisms, implications, and applications for trophic ecology. Ecosphere, 7(12), 4772–26.

[ece35080-bib-0050] Metcalfe, N. B. , Taylor, A. C. , & Thorpe, J. E. (1995). Metabolic rate, social status and life‐history strategies in Atlantic salmon. Animal Behavior, 49, 431–436. 10.1006/anbe.1995.0056

[ece35080-bib-0051] Metcalfe, N. B. , Van Leeuwen, T. E. , & Killen, S. S. (2016). Does individual variation in metabolic phenotype predict fish behavior and performance? Journal of Fish Biology, 88, 298–321. 26577442 10.1111/jfb.12699 PMC4991269

[ece35080-bib-0052] Milligan, C. L. , & Wood, C. M. (1987). Effects of strenuous activity on intracellular and extracellular acid‐base status and H^+^ exchange with the environment in the inactive, benthic starry flounder *Platichthys stellatus* . Physiological Zoology, 60, 37–53.

[ece35080-bib-0053] Morozov, S. , Leinonen, T. , Merilä, J. , & McCairns, R. J. S. (2018). Selection on the morphology‐physiology‐performance nexus: Lessons from freshwater stickleback morphs. Ecology and Evolution, 8, 1286–1299. 10.1002/ece3.3644 29375798 PMC5773335

[ece35080-bib-0054] Morrow, J. E. (1980). The freshwater fishes of Alaska. Anchorage, AK: Alaska Northwest Publishing Co.

[ece35080-bib-0055] Munroe, T. A. (2015). Systematic diversity of the Pleuronectiformes In Gibson R. N., Nash R. D. M., Geffen A. J., & Van der Veer H. W. (Eds.), Flatfishes: Biology and exploitation (pp. 13–51). West Sussex, UK: Wiley Blackwell.

[ece35080-bib-0056] Nabeta, K. K. (2009). The type 1 antifreeze protein gene family in Pleuronectidae. MSc 131. Kingston, ON: Department of Biomedical and Molecular Sciences, Queen's University.

[ece35080-bib-0057] Olla, B. L. , Samet, C. E. , & Studholme, A. L. (1972). Activity and feeding behavior of the summer flounder (*Paralichthys dentatus*) under controlled laboratory conditions. Fishery Bulletin, 71, 1127–1136.

[ece35080-bib-0058] Orcutt, H. G. (1950). The life history of the starry flounder *Platichthys stellatus* (Pallus). California Department of Fish and Game Fish Bulletin, 78, 4772–64.

[ece35080-bib-0059] Peake, S. , McKinley, R. S. , & Scruton, D. A. (2005). Swimming performance of various freshwater Newfoundland salmonids relative to habitat selection and fishway design. Journal of Fish Biology, 51, 710–723. 10.1111/j.1095-8649.1997.tb01993.x

[ece35080-bib-0060] Plaut, I. (2001). Critical swimming speed: Its ecological relevance. Comparative Biochemistry and Physiology Part A, 131, 41–50. 10.1016/S1095-6433(01)00462-7 11733165

[ece35080-bib-0061] Policansky, D. (1982). The asymmetry of flounders. Scientific American, 246, 116–122. 10.1038/scientificamerican0582-116

[ece35080-bib-0062] Priede, I. G. , & Holliday, F. G. T. (1980). The use of a new tilting tunnel respirometer to investigate some aspects of metabolism and swimming activity of the plaice (*Pleuronectes platessa* L.). Journal of Experimental Biology, 85, 295–309.

[ece35080-bib-0063] Reimchen, T. E. (1994). Predators and evolution in threespine stickleback In Bell M. A., & Foster S. A. (Eds.), Evolution of the threespine stickleback (pp. 240–273). Oxford, UK: Oxford University Press.

[ece35080-bib-0064] Reimchen, T. E. , Bergstrom, C. A. , & Nosil, P. (2013). Natural selection and the adaptive radiation of Haida Gwaii stickleback. Evolution Ecology Research, 15, 241–269.

[ece35080-bib-0065] Rice, A. N. , & Hale, M. E. (2010). Roles of locomotion in feeding In Domenici P., & Kapoor B. G. (Eds.), Fish locomotion: An eco‐ethological perspective (pp. 171–199). Enfield, NH: Science Publishers.

[ece35080-bib-0066] Rupia, E. J. , Binning, S. A. , Roche, D. G. , & Lu, W. (2016). Fight‐flight or freeze‐hide? Personality and metabolic phenotype mediate physiological defence responses in flatfish. Journal of Animal Ecology, 85, 927–937. 10.1111/1365-2656.12524 27044558

[ece35080-bib-0067] Russo, T. , Pulcini, D. , Costantini, D. , Pedreschi, D. , Palamara, E. , Boglione, C. , … Mariani, S. (2012). “Right” or “wrong”? Insights into the ecology of sidedness in European flounder, *Platichthys flesus* . Journal of Morphology, 273, 337–346. 10.1002/jmor.11027 22025394

[ece35080-bib-0068] Schmidt‐Nielsen, K. (1997). Animal physiology: Adaptation and environment, 5th ed Cambridge, NJ: Cambridge University Press.

[ece35080-bib-0069] Shao, C. , Bao, B. , Xie, Z. , Chen, X. , Li, B. o. , Jia, X. , … Chen, S. (2017). The genome and transcriptome of Japanese flounder provide insights into flatfish asymmetry. Nature Genetics, 49, 119–124. 10.1038/ng.3732 27918537

[ece35080-bib-0070] Shine, R. , Ambariyanto , Harlow, P. S. , & Munpuni (1998). Ecological divergence among sympatric colour morphs in blood pythons, *Python brongersmai* . Oecologia, 116, 113–119. 10.1007/s004420050569 28308515

[ece35080-bib-0071] Sinervo, B. , & Svensson, E. (2002). Correlational selection and the evolution of genomic architecture. Heredity, 89, 329–338. 10.1038/sj.hdy.6800148 12399990

[ece35080-bib-0072] Svensson, E. , Sinervo, B. , & Comendant, T. (2001). Condition, genotype‐by‐environment interaction, and correlational selection in lizard life‐history morphs. Evolution, 55, 2053–2069. 10.1111/j.0014-3820.2001.tb01321.x 11761065

[ece35080-bib-0073] Taylor, E. B. , & McPhail, J. D. (1986). Prolonged and burst swimming in anadromous and freshwater threespine stickleback, *Gasterosteus aculeatus* . Canadian Journal of Zoology, 64, 416–420.

[ece35080-bib-0074] Wainwright, P. C. (1991). Ecomorphology: Experimental functional anatomy for ecological problems. Integrative and Comparative Biology, 31, 680–693.

[ece35080-bib-0075] Walker, J. A. , Ghalambor, C. K. , Griset, O. L. , McKenney, D. , & Reznick, D. N. (2005). Do faster starts increase the probability of evading predators? Functional Ecology, 19, 808–815.

[ece35080-bib-0076] Walsh, S. J. , & Morgan, J. (2004). Observations of natural behavior of yellowtail flounder derived from data storage tags. ICES Journal of Marine Science, 61, 1151–1156.

[ece35080-bib-0077] Webb, P. W. (1976). The effect of size on the fast‐start performance of rainbow trout *Salmo gairdneri* and a consideration of piscivorous predator‐prey interaction. Journal of Experimental Biology, 65, 157–177. 993700 10.1242/jeb.65.1.157

[ece35080-bib-0078] Webb, P. W. (1978). Temperature effects on acceleration of rainbow trout *Salmo gairdneri* . Journal of the Fisheries Research Board of Canada, 35, 1417–1422.

[ece35080-bib-0079] Webb, P. W. (1981). The effect of the bottom on the fast start of flatfish *Citharichthys stigmaeus* . Fishery Bulletin, 79, 271–276.

[ece35080-bib-0080] Webb, P. W. (1984). Body form, locomotion and foraging in aquatic vertebrates. American Zoologist, 24, 107–120. 10.1093/icb/24.1.107

[ece35080-bib-0081] Webb, P. W. (1988). Steady swimming kinematics of tiger musky, an escociform accelerator, and rainbow trout, a cruiser generalist. Journal of Experimental Biology, 138, 51–69.

[ece35080-bib-0082] Wei, F. , Chen, J. , Chen, X. , & Bao, B. (2017). Comparative analysis of the neurula transcriptomes of two species of flatfishes: *Platichthys stellatus* and *Paralichthys olivaceus* . Gene, 596, 147–153. 10.1016/j.gene.2016.10.020 27751815

[ece35080-bib-0083] Winger, P. D. , He, P. , & Walsh, S. J. (1999). Swimming endurance of American plaice (*Hippoglossoides platessoides*) and its role in fish capture. ICES Journal of Marine Science, 56, 252–265. 10.1006/jmsc.1999.0441

[ece35080-bib-0084] Wood, C. M. , McMahon, B. R. , & McDonald, D. G. (1977). An analysis of changes in blood pH following exhausting activity in the starry flounder, *Platichthys stellatus* . Journal of Experimental Biology, 69, 173–185. 908908 10.1242/jeb.69.1.173

[ece35080-bib-0085] Yan, G.‐J. , He, X.‐K. , Cao, Z.‐D. , & Fu, S.‐J. (2013). An interspecific comparison between morphology and swimming performance in cyprinids. Journal of Evolutionary Biology, 26, 1802–1815. 23869541 10.1111/jeb.12182

